# Scintigraphic Evaluation of the Stump Region After Extremity Amputation and the Effect of Scintigraphy on Treatment

**DOI:** 10.14740/jocmr2459w

**Published:** 2016-01-26

**Authors:** Murat Sadic, Hasan Ikbal Atilgan, Aylin Baskin, Alev Cinar, Gokhan Koca, Koray Demirel, Aylin Comak, Sinem Ozyurt, Sule Yildirim, Meliha Korkmaz

**Affiliations:** a Department of Nuclear Medicine, Ministry of Health Ankara Training and Research Hospital, Ankara, Turkey; b Division of Nuclear Medicine, Ministry of Health Kahramanmaras Necip Fazil City Hospital, Kahramanmaras, Turkey; c Division of Nuclear Mecicine, Ministry of Health Sami Ulus Children Hospital, Ankara, Turkey; d Department of Nuclear Medicine, Dr. Abdurrahman Yurtaslan Ankara Oncology Training and Research Hospital, Ankara, Turkey

**Keywords:** Stump region, Osteomyelitis, Tc-99m HDP scintigraphy

## Abstract

**Background:**

We evaluated the stump region with scintigraphy and compared the correlation of treatment modalities and scintigraphic results.

**Methods:**

Sixty-eight cases with extremity amputation were included in the study. Amputation applied cases underwent four-phase Tc-99m hydroxymethylene diphosphonate scintigraphy. Groups were performed according to the scanning time after amputation and amputation regions. After scintigraphic evaluation, results were recorded into five groups: osteomyelitis, soft-tissue infection, reactive changes secondary to surgery, chronic osteomyelitis, and normal. Post-surgical treatment modalities of the patients were determined and compared with scintigraphic results.

**Results:**

In the scintigraphic evaluation of stump regions of the 68 amputated cases, 34 patients had acute osteomyelitis, one had chronic osteomyelitis, 16 had soft-tissue infection, and eight had changes secondary to the surgery. Nine of 68 cases had normal scintigraphic features. In the scintigraphic evaluation, 43 patients took antibiotic treatment and 16 had surgery. There was a strong correlation between scintigraphic results and treatment approach (P < 0.0001, r = 0.803) by means of preferred therapy and effectiveness of the therapy according to the scintigraphic results. Scintigraphy need increases with age after amputation and a negative correlation between patient age and scintigraphic need was found (P < 0.02, r = -0.339). There was no pathology in the follow-up in the cases that were scintigraphically normal.

**Conclusion:**

Bone scintigraphy is a cost-effective, non-invasive, and efficient method that directs treatment in the evaluation of the stump region after amputation.

## Introduction

Extremity amputations are frequently performed procedures for many reasons and in different levels. Trauma, burns, diabetes, and peripheral vascular diseases are the common causes for the etiology [1, 2]. Infection progression after neuropathy or ischemia causes amputation regardless of the etiology. The major cause (50-70%) of non-traumatic limb amputations is diabetic foot infections [2]. The probability of lower extremity amputation in patients with diabetes is around 5-15% and probability of the other extremity amputation is higher. One type of microorganism (*Staphylococcus aureus* is the most common) is often seen in the newly formed wounds, whereas polymicrobial, resistant strains are found in chronic, recurrent wounds [2-6]. Anaerobes participate in the presence of ischemia and gangrene. After amputation, in the operation region, infection is affected by the width of surgery, patient’s general status, comorbidities, and postoperative care. Infection should be diagnosed immediately, but laboratory methods may be misleading due to the surgery and broad-spectrum prophylactic antibiotic therapy. Digital X-ray may not reflect the changes in the early period. Diagnosis and treatment of osteomyelitis is an important problem. In case of exposed bone in the wound, osteomyelitis should be ruled out. When the other methods are insufficient for diagnosis, scintigraphy may show increased perfusion and hyperemia due to inflammation in the early period, and help soft tissue-bone distinction. The diagnosis is usually started by the first-line method digital X-ray, but in suspected cases, magnetic resonance imaging (MRI) and/or scintigraphy is used. Bone biopsy is the gold standard for the diagnosis [6-11]. In our literature review, we did not find any study about the scintigraphic evaluation of the stump region after extremity amputation. We aimed to evaluate the effect of bone scintigraphy in the evaluation of the stump region and the contribution of scintigraphy to the treatment modality.

## Materials and Methods

**Figure 1:**
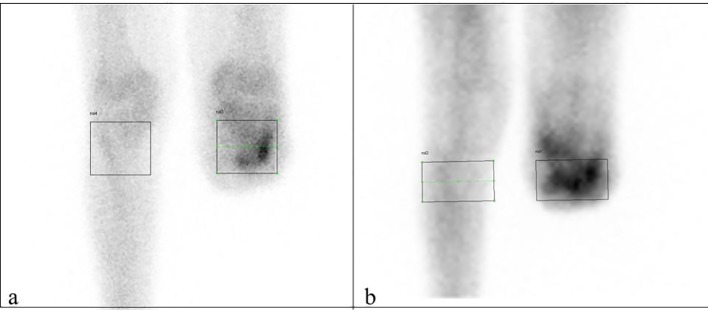
Late (a) and early (b) phase ROI samples.

Sixty-eight patients (51 male and 17 female) with mean age of 53.7 ± 15.8 years (min: 2; max: 82) that were referred to the Nuclear Medicine Department of Ankara Training and Research Hospital for the evaluation of infection after amputation between 2003 and 2013 were included in the study. The study protocol was approved by the institutional review board of the Local Ethics Committee of our hospital. All the patients had digital X-ray, superficial tissue ultrasonography, white blood cell count, sedimentation and C-reactive protein levels before the four-phase bone scintigraphy. The study was prepared retrospectively and 27 patients had MRI. Four-phase bone scintigraphy with Tc-99m hydroxymethylene diphosphonate (HDP) was given to all patients. The bilateral amputated extremity region was placed in the field of the collimator. Immediately after injection, dynamic early static (blood pool phase) images were taken, and late static images 3 and 24 h later. After 3 h, anterior and posterior whole body scans were performed. The images were taken with a single detector gamma camera (Siemens e.cam/e.soft gamma camera, USA) using a low-energy general-purpose collimator, with a range of 140 keV to give a 20% window. Patients were divided into four groups according to the time of amputation (0 - 3 months, 3 months, 1 - 3 years, and more than 3 years) and amputation region (foot, toes, knee, femur, and upper extremity). Evaluation was made visually by comparing with the normal opposite extremity (Fig. 1). Results were classified into five groups: osteomyelitis, soft-tissue infection, reactive changes secondary to surgery, and chronic osteomyelitis. For the quantitative analysis, rectangular region of interests (ROIs) were drawn to the lesion and the normal side in the early and late static images and calculated as the formula “count of ROI in lesion side/count of ROI in normal side”. In the follow-up of the patients, culture results in lesions and the treatment modality were analyzed and compared statistically.

### Statistical analysis

Data analysis was performed using Statistical Package for Social Sciences for Windows software (SPSS version 17.0, SPSS Inc., Chicago, IL, USA). Compliance with the normal distribution of variables was evaluated visually (histogram and probability plots) and by analytic methods (Kolmogorov-Smirnov/Shapiro-Wilk tests). Descriptive analyses are expressed as mean ± standard deviation. Student’s *t*-test was used to compare the ROIs of the lesion and the normal side, and P < 0.05 was accepted as statistically significant.

## Results

As shown in Table 1, diabetes-related vascular disorders were present in 70.6% of the 68 patients, trauma caused by traffic accidents in 27.9%, and compartment in 1.5%. The mean age was 53.7 years, the youngest patient was 2 years old with an amputated arm due to compartment syndrome, and the oldest patient was 82 years old with an amputation due to diabetic foot. The scintigraphy was performed on average 8 months after the amputation; 75% of the patients were male and 25% were female. Pain was present in all patients, redness and warmth in 42 patients, and draining fistula in 11 patients. The mean value of C-reactive protein was 1.8 (0 - 0.4), and white blood cell level and erythrocyte sedimentation rate were slightly elevated. Thirty-four patients had acute osteomyelitis, one had chronic osteomyelitis, 16 had soft-tissue infection, and eight had changes secondary to surgery. Scintigraphic findings were within normal limits in nine of 68 patients. The lesion regions were toes, femur, upper extremity, tibia, and foot respectively by frequency. Forty-three patients had antibiotic therapy and 16 had surgical procedures. Culture results of the patients before the antibiotic therapy were used for the clarification of the diagnosis. Antibiotic therapy was started to the patients that had osteomyelitis diagnoses in the follow-up and therapy was changed only according to their antibioticograms. One of eight patients had surgery, two had antibiotic therapy, and five had no treatment in the group that had reported as changes secondary to surgery with the scintigraphy. Two patients had hyperbaric oxygen therapy and had the effect of this therapy. There was a strong positive correlation between the scintigraphic results and treatment modality (P < 0.0001, r = 0.803). The need of scintigraphic imaging increases with age. There was no pathology in the follow-up with normal scintigraphy. Lesion/control ratio was 2.34 in the early phase and 3.47 in the late phase in the acute osteomyelitis group, and 1.03 and 1.35 respectively in the normal group (Table 2).

**Table 1:** Demographic Characteristics of Patients

Variables	n (%)
Gender	
Male	51 (75%)
Female	17 (25%)
Cause of amputation	
Trauma (accident)	19 (27.9%)
Diabetes mellitus	48 (70.6%)
Compartment syndrome	1 (1.5%)
Amputation region	
Toes	20 (29.4%)
Foot	3 (4.4%)
Tibia/under knee	12 (17.6%)
Femur	17 (25%)
Upper extremity	16 (23.5%)
Result of scintigraphy	
Osteomyelitis	34 (50%)
Soft-tissue infection	16 (23.5%)
Secondary to surgery	8 (11.8%)
Chronic osteomyelitis	1 (1.5%)
Normal	9 (13.2%)
Treatment modality	
Antibiotics	41 (60.3%)
No treatment	9 (13.2%)
Surgery	16 (23.5%)
Hyperbaric oxygen therapy	2 (3%)

**Table 2:** Early and Late Mean Counts in the Scintigraphic Images

Diagnosis	n	Early ratio	P value	Late ratio	P value
Osteomyelitis	34	2.34 ± 1.67	0.025	3.47 ± 1.95	0.003
Soft tissue infection	16	1.55 ± 0.89	0.12	2.3 ± 1.04	0.017
Secondary to surgery	8	1.47 ± 0.85	0.19	2.77 ± 1.41	0.025
Normal	9	1.03 ± 0.43		1.35 ± 0.49	

Eleven of the 27 patients were evaluated as normal with MRI, 12 patients had bone-marrow edema in the proximal part of the amputation region and soft-tissue edema, and four patients had thickness and inflammatory changes in subcutaneous tissue and skin.

## Discussion

**Figure 2:**
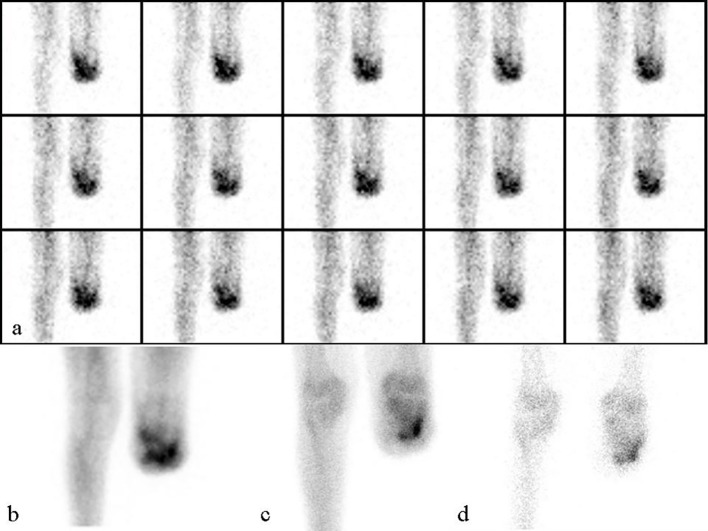
A 71-year-old male underwent left under-knee amputation 6 months ago, referred with a draining wound for bone scintigraphy. Scintigraphy was reported as osteomyelitis: (a) perfusion images; (b) blood pool images; (c) 3 h late anterior knee static images; (d) 24 h late knee static anterior images.

**Figure 3:**
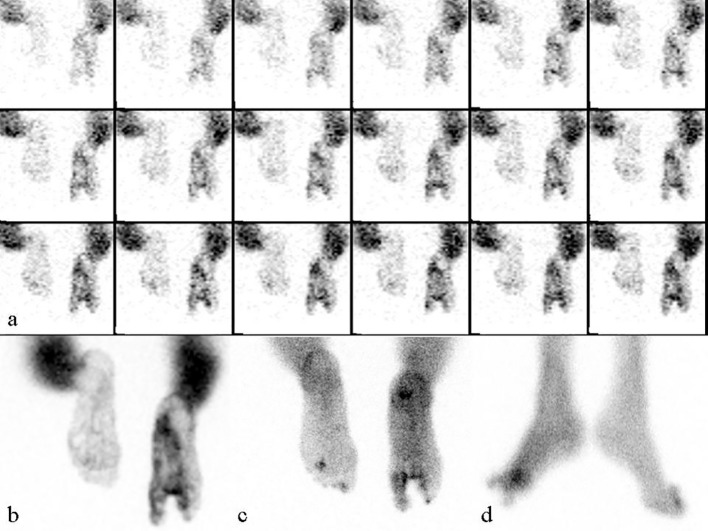
A 44-year-old female underwent second and third right toe amputation due to diabetes 1 year ago. One month after the amputation, bone scintigraphy was taken for the draining wound. After the antibiotic therapy, symptoms decreased. After recurrence of the draining wound in the stump region, bone scintigraphy was performed again. In the comparative evaluation, the images were reported as chronic osteomyelitis: (a) perfusion images; (b) blood pool images; (c) 3 h static images; (d) 3 h lateral static images.

The amputation level of the non-functional and devitalized extremity is important for the patient’s quality of life and level of functionality. The most common causes of amputation are trauma, diabetic vasculopathy, peripheral vascular disease, and tumors in the classical publications [12]. In our study, the most common causes are diabetic vasculopathy and trauma as in previous publications (Fig. 2). The risk of local recurrence is the age that may affect the patient’s general status as well as the width of the surgical margin [13-17]. Coupland et al mentioned the importance of the amputation level and postoperative care [17], because infection in the stump region of below-knee amputations may proceed to above-knee amputation. In our study, the most common level of the amputation was the femur (Fig. 3).

Osteomyelitis in the amputated stump region is hard to treat, and reduces the quality of life, and recurrence is common despite the various treatments. Osteomyelitis often develops in direct contact with soft-tissue infection [18-21]. Good results can be obtained after the surgical debridement of infected and necrotic tissues and bones, and with good long-term antibiotic treatment. If postoperative stump region infection occurs within 3 months, it is called as early-stage infection, delayed-stage infection between 3 and 24 months, and late-stage infection after 24 months. In our study, 66% of patients were investigated for late-stage infection. Prevention of osteomyelitis is easier and cheaper than the treatment of osteomyelitis. Osteomyelitis developed after orthopedic surgery tends to be chronic. Antibiotic prophylaxis is used successfully in orthopedic surgical procedures for the prevention of surgical infections. There are two staging systems in adult osteomyelitis. The first is the anatomical and physiological staging made by Cierny and Mader [22-25]. Only antibiotic therapy is enough in stage 1, whereas stage 3 osteomyelitis requires aggressive surgical debridement, long-term antibiotic therapy, and orthopedic reconstruction [9]. The second staging system by Lew and Waldvogel considers stage (acute or chronic), mechanism of infection, and vascular disorders [10]. Radical cleaning of necrotic tissue, filling the dead gaps, and long-term appropriate antibiotic therapy are the main principles in the treatment of osteomyelitis [4, 5]. Surgery is essential in patients with chronic and acute osteomyelitis that forms abscesses. The aim is the debridement of sequestered, infected, and scarred soft tissues and to form a good vascular bed. Inadequate debridement likely causes relapse and chronicity. Soft tissue and bone defects that need reconstruction may persist after large resections. Beside the bactericidal effect of the antibiotics, penetration to the bone tissue is highly important. When diagnosis or even the possibility of infection occurs after surgical procedures, antibiotic treatment should be started as soon as possible. Recently, hyperbaric oxygen therapy, especially in diabetes mellitus, has also been used in the treatment of osteomyelitis. Some publications mentioned the effectiveness of this treatment [26]. Neutrophils can not kill pathogens and fibroblasts can not make angiogenesis, and this causes delayed wound healing in inadequate oxygen levels. This therapy increases the oxygen saturation and increases the fibroblast functions, and some anaerobic bacteria cannot survive. In our study, two patients had this therapy and saw the benefit of this method.

Digital X-ray is the first imaging modality for the evaluation of osteomyelitis, sensitivity is 54% and specificity is 68% [27]. Ultrasonography (USG) is a cheap, easy available technique but it is dependent on the user, and the ultrasonographic sound waves cannot penetrate the cortical bone. The main finding is periosteal elevation, soft-tissue abscess, and edema. In our study, all patients underwent USG and all but 11 patients had edema in the lesion side. Computed tomography was not used in our study but its sensitivity is around 67% and specificity is 50%. MRI has high resolution: in the early diagnosis, its sensitivity is 82-100% and specificity is 75-96% for the showing of the effected tissue [26-28]. Medullary hypointensity, inflammation, hyperintensity due to edema, radiolucent abscess, and in chronic osteomyelitis non-vascularized hypointense fibrotic scar tissue in bone marrow can be seen in the lesion side. Eleven of the 27 patients that underwent MRI were evaluated as normal, 12 patients had bone-marrow edema in the proximal part of the amputation region and soft-tissue edema, and four patients had thickness and inflammatory changes in subcutaneous tissue and skin. The four-phase bone scintigraphy has high sensitivity, is relatively inexpensive, is highly accessible, and is an important method in early diagnosis, but advanced imaging methods may be needed due to its low specificity. The specificity can be increased with quantitative evaluation. Acute osteomyelitis was in 34 patients (50%), soft-tissue infection in 16, chronic osteomyelitis in one, and changes secondary to surgery in eight patients with scintigraphy. In the early phase of infection, pain, effusion, swelling, redness, and warmth are seen and draining fistula may be seen. In the late phase, the main symptom is pain. In the current study, pain was present in all patients, redness and warmth in 42 patients, and draining fistula in 11 patients. In the first 10 days, digital X-rays are not useful, because any findings cannot be seen. Later, periosteal reaction, lytic bone lesions, and cortical destruction may occur. Nowadays, bone scintigraphy and MRI are relied on more in the diagnosis of osteomyelitis. Increased activity is seen within 24 - 48 h of onset of symptoms. Bone scintigraphy has over 90% sensitivity and 70-90% specificity in acute hematogenous osteomyelitis [21]. The sensitivity and specificity of MRI are close to each other [15]. The sensitivity of MRI in osteomyelitis of the peripheral skeleton is not different from leukocyte scintigraphy of combined bone and gallium scintigraphy. MRI has high sensitivity and specificity in the diagnosis of chronic osteomyelitis [22-25]. The high resolution of MRI is very useful for the differentiation of bone and soft-tissue infection, whereas it is difficult with radionuclide methods. On the other hand, whole body imaging is the advantage of bone scintigraphy. The dynamic phase reflects the perfusion and blood-pool phase reflects the activity extravasation around the tissue. Focal increased perfusion, hyperemia, and increased uptake are expected classically with three-phase bone scintigraphy. Increased activity accumulation that is seen in the late phase is not specific to infection and may be seen in tumors, fracture, and arthropathy. For this reason, gallium-67 and Tc-99m human immune globulin, or indium-111-labeled leukocytes are often used as complementary techniques [15, 16]. Gallium-67 scintigraphy has 83% sensitivity and 79% specificity; indium-111-labeled leukocyte scintigraphy has 83-100% sensitivity and 90% specificity [4, 16]. Osteomyelitis can be confirmed with biopsy, but at least in 48 h. As infection progresses, blood flow will be reduced and technetium-labeled scintigraphy may give false-negative results [14].

In our study, four-phase Tc-99m HDP scintigraphy was performed on all 68 patients. Thirty-four patients had acute osteomyelitis, one patient had chronic osteomyelitis, and 16 patients had soft-tissue infection. If the treatment modality generated according to the scintigraphic evaluation, the clinical outcome of the patients improves. In other words, none of the infected patients were misdiagnosed, but nine patients that had been evaluated as normal and eight patients as changes secondary to surgery developed infection.

### Conclusion

Bone scintigraphy is an effective and accurate imaging technique that directs the treatment modality. Changes in the treatment modality according to the scintigraphic evaluation have high value.
